# Phenotypic Selection of Dairy Cattle Infected with Bovine Leukemia Virus Demonstrates Immunogenetic Resilience through NGS-Based Genotyping of BoLA MHC Class II Genes

**DOI:** 10.3390/pathogens11010104

**Published:** 2022-01-15

**Authors:** Chaelynne E. Lohr, Kelly R. B. Sporer, Kelsey A. Brigham, Laura A. Pavliscak, Matelyn M. Mason, Andrew Borgman, Vickie J. Ruggiero, Tasia M. Taxis, Paul C. Bartlett, Casey J. Droscha

**Affiliations:** 1 CentralStar Cooperative, Lansing, MI 48910, USA; chaelynne.lohr@mycentralstar.com (C.E.L.); kelly.sporer@mycentralstar.com (K.R.B.S.); kelsey.brigham@mycentralstar.com (K.A.B.); Laura.Pavliscak@mycentralstar.com (L.A.P.); masonm19@msu.edu (M.M.M.); 2 Borgman Consulting Group LLC, Alma, MI 48801, USA; ndrwsbrgmn@gmail.com; 3 College of Veterinary Medicine, Michigan State University, East Lansing, MI 48824, USA; woodsvi1@msu.edu (V.J.R.); Bartle16@msu.edu (P.C.B.); 4 Department of Animal Science, College of Large Animal Clinical Sciences, Michigan State University, East Lansing, MI 48824, USA; taxistas@msu.edu

**Keywords:** bovine leukemia virus, ELISA, proviral load, BoLA, MHC Class II, sequence-based typing, molecular diagnostics, qPCR, immunogenetics, disease resilience, dairy science

## Abstract

Characterization of the bovine leukocyte antigen (BoLA) DRB3 gene has shown that specific alleles associate with susceptibility or resilience to the progression of bovine leukemia virus (BLV), measured by proviral load (PVL). Through surveillance of multi-farm BLV eradication field trials, we observed differential phenotypes within seropositive cows that persist from months to years. We sought to develop a multiplex next-generation sequencing workflow (NGS-SBT) capable of genotyping 384 samples per run to assess the relationship between BLV phenotype and two BoLA genes. We utilized longitudinal results from milk ELISA screening and subsequent blood collections on seropositive cows for PVL determination using a novel BLV proviral load multiplex qPCR assay to phenotype the cows. Repeated diagnostic observations defined two distinct phenotypes in our study population, ELISA-positive cows that do not harbor detectable levels of provirus and those who do have persistent proviral loads. In total, 565 cows from nine Midwest dairy farms were selected for NGS-SBT, with 558 cows: 168 BLV susceptible (ELISA-positive/PVL-positive) and 390 BLV resilient (ELISA-positive/PVL-negative) successfully genotyped. Three BoLA-DRB3 alleles, including one novel allele, were shown to associate with disease resilience, *009:02, *044:01, and *048:02 were found at rates of 97.5%, 86.5%, and 90.3%, respectively, within the phenotypically resilient population. Alternatively, DRB3*015:01 and *027:03, both known to associate with disease progression, were found at rates of 81.1% and 92.3%, respectively, within the susceptible population. This study helps solidify the immunogenetic relationship between BoLA-DRB3 alleles and BLV infection status of these two phenotypic groupings of US dairy cattle.

## 1. Introduction

Bovine leukemia virus (BLV) belongs to the *deltaretrovirus* genus within the *Retroviridae* family and is the cause of enzootic bovine leukosis (EBL), the most common neoplastic disease in dairy cattle. Since the recognition of BLV in the late 1960s, within-herd disease prevalence in the United States has risen from 10 to over 40%, with 92% of farms having at least one seropositive cow by enzyme-linked immunosorbent assay (ELISA) [1]. BLV targets the host’s B lymphocytes and is propagated through clonal expansion of immature B cells following infection. Most BLV-infected cattle are asymptomatic, yet 30% of ELISA-positive cattle develop persistent lymphocytosis (PL), defined as an increase in blood lymphocyte concentration caused by B-cell proliferation for at least three months [2]. Only a small number of cattle with PL (1–5%) develop B lymphocyte lymphoma; however, PL cattle have an increased probability of transmitting BLV to their herdmates, incurring a persistent economic burden to dairy producers [3,4].

One common method for BLV diagnosis is the detection of anti-BLV antibodies, often anti-BLV gp51 or p24 antibodies, by ELISA on milk or serum. Detection of BLV proviral DNA in whole blood is most often accomplished through quantitative PCR (qPCR). Multiplex qPCR allows for the direct comparison of BLV proviral DNA copies with host DNA simultaneously. Our group defines proviral load (PVL) as the relative concentration of BLV proviral DNA to host DNA detected within a blood-derived genomic DNA extract (gDNA). PVL is used as a measure of the level of infectiousness, as done within other retroviruses such as the closely related human T-cell lymphotropic virus (HTLV) [5]. We have found that a proportion of cows are ELISA-positive but do not have a detectable PVL and do not progress in disease status. One possible explanation for these differing phenotypes is the contribution of host genetics, specifically within genomic loci directly tied to the humoral immune response.

Genetic diversity contributes to the variability in the adaptive and innate immune system’s ability to recognize and neutralize divergent invading pathogens [6]. The major histocompatibility complex (MHC) is a cluster of genes essential for proper immune system function in higher order mammals [7,8]. MHC class II genes are involved in pathogen antigen processing and transport via the endo-lysosomal vesicular system as well as extracellular presentation, of non-self-derived peptides. Presentation of foreign-derived peptides is largely dependent on structural features of the binding groove of specific MHC allelic variants. The polymorphic nature of MHC class II genes is an important genetic feature for individual animals within populations to collectively mount a robust and diverse response to a common emerging pathogen. Unique combinations of heterodimeric antigen receptors within individual animals allow for proper antigen recognition and presentation. The MHC region in humans is called the human leukocyte antigen (HLA) and has been associated with resistance or susceptibility to many diseases, including but not limited to rheumatoid arthritis [9], type 1 diabetes [10], and Hodgkin’s disease [11]. Additionally, the bovine leukocyte antigen (BoLA), the MHC region in cattle, has been linked to progression of mastitis, dermatophilosis, BLV, and more [7].

Several species have been documented as being susceptible or resistant to specific diseases and pathogens, including sheep, chicken, pigs, goats, and cattle [12]. Pedigree analysis from 1970 showed that bovine lymphosarcoma and PL segregate along family lineages, providing the first evidence for genetic influence on susceptibility to BLV [13]. Originally, serologically defined BoLA class I alleles were found to be associated with BLV phenotype [14]; however, MHC class II alleles were later identified as playing a stronger role in BLV outcome in cattle [15]. The BoLA MHC class II gene DRB3 is highly polymorphic with 384 identified alleles to date [16] and is the only functional locus within the DR genes and is thus the strongest expressed gene within this cluster [17]. In contrast, there are numerous functional DQ genes [18]. MHC class II molecules have peptide chains with two external domains, the distal domain forms the peptide binding cleft, whereas the second domains are proximal to the cell membrane [7]. The second exon of the MHC II BoLA-DRB3 and DQA1 alleles encode for the extracellular portion of the molecule, whereby allelic diversity provides individual cow’s antigen-presenting cells with unique immunity to various pathogens. Although numerous alleles have been classified as leading to a resistant or susceptible BLV phenotype, DRB3*009:02 has been most notably associated with the resistant phenotype [15,19,20,21,22,23]. More recently, polymorphisms within the DRB3 gene have been identified as having a stronger association with BLV phenotype than the DQA1 gene [24].

Our group has performed test and cull BLV eradication field trials for over four years, through utilization of whole-herd BLV profiles by ELISA screening and follow-up PVL testing from the blood of ELISA-positive cows. The resulting sample repository and diagnostic database from these field trials has allowed for retrospective phenotypic-driven genetic analysis. In this study, we set out to develop a high-throughput multiplex NGS-based typing method for the accurate and efficient characterization of two BoLA loci known to be associated with BLV status. Our novel approach of BLV phenotyping prior to BoLA genotyping strongly agrees with previously published evidence that specific BoLA-DRB3 alleles associate with BLV disease progression.

## 2. Results

### 2.1. Phenotype Determination Using Longitudinal Diagnostic Testing of BLV-Infected Cows

The objective of the BLV eradication field trials were to assess whether whole herd screening and selective culling approaches within commercial dairy herds effectively reduced overall herd prevalence over time [25,26]. At each timepoint, we observed that 5–15% of ELISA-positive cows did not harbor detectable levels of provirus as determined by the SS1 qPCR BLV PVL Assay (data not shown). These phenotypes persisted from several months to years, depending on the culling decisions of the producer. The SS1 qPCR BLV PVL Assay has been extensively characterized for its specificity and sensitivity, with a limit of detection of 10 copies of BLV proviral DNA [25,27] (Figure S1). After screening over 14,000 cows for BLV antibodies in their milk and testing 4000 for PVL over four years (Table 1), two distinct phenotypic groups were identified; ELISA-positive cows without a PVL and those with detectable levels of provirus.

**Figure 1:**
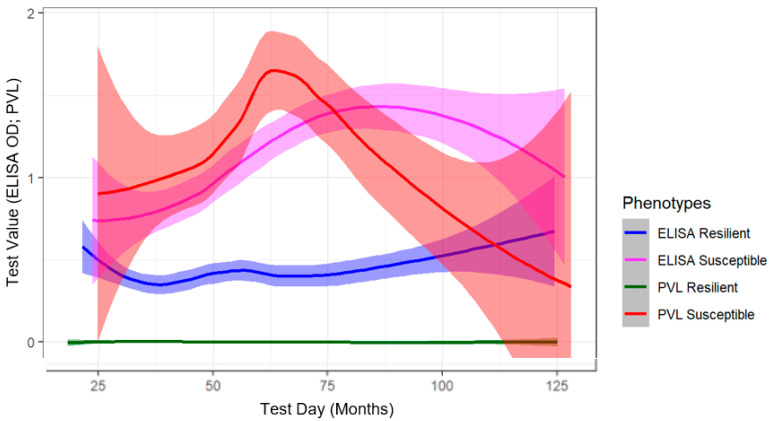
Longitudinal DHI milk ELISA and blood PVL modeling of BLV susceptible and resilient cows selected for genotyping. All available DHI milk ELISA and blood derived PVL values were modeled over the age of each cow. 246 resilient DHI milk ELISA (blue), 384 resilient PVL (green), 116 susceptible ELISA (pink), and 169 susceptible (red) cow BLV diagnostic histories are represented here. Number of times individual cows were ELISA tested varies. Susceptible: 1× = 18, 2× = 35, 3× = 67, 4× = 2 and 5× = 2. Resilient: 1× = 96, 2× = 98, 3× = 83, 4× = 8 and 5× = 2. Line backdrop represents 95% confidence interval.

For this study, cows that had a positive BLV ELISA result, but no detectable level of provirus, were considered phenotypically resilient to BLV. In contrast, cows with consistent detectable levels of BLV provirus were classified as BLV susceptible. Phenotypes were determined using one-five timepoints, dependent on the availability of data due to the specific field trial and producer culling decisions. To assess all cows per cohort throughout the four-year field trials, test values were plotted against the age of the cow at testing day (Figure 1). Using geom_smooth within the library ggplot2, we created a line of best fit from the scatter of diagnostic values over time via the loess spline curve method, with shaded regions representing a 95% confidence interval [28]. Loess is a localized non-parametric regression approach to assess trends over time (http://r-statistics.co/Loess-Regression-With-R.html, R package V 1.4.1106, accessed on 25 June 2021). Milk ELISA testing preceded PVL testing by approximately one month in six-month or one-year intervals, depending on herd and active field trial. Dairy Herd Information Association (DHI) milk ELISA optical density (OD) values and SS1 qPCR BLV PVL Assay values fall within the same range allowing for simultaneous visualization of the disease status of all cows over their lifespan. A subset of cows within this analysis were screened via serum BLV ELISA rather than DHI milk ELISA, depending on the field trial. While their ELISA status was maintained and used for phenotype determination, serum ELISA values were omitted from the analysis for Figure 1, due to the difference in range between serum and milk ELISA values.

### 2.2. Development of Multiplex Sequencing Approach

**Figure 2:**
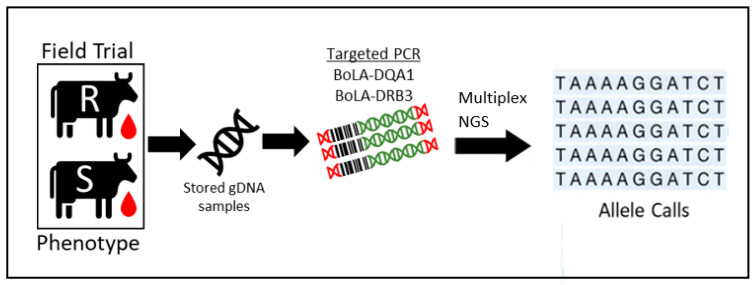
NGS-SBT experimental workflow. Cows were selected based on BLV phenotype, and stored gDNA samples were used for targeted amplification of the BoLA DQA1 and DRB3 genes.

We sought to develop a multiplex next generation sequence-sequence based typing (NGS-SBT) approach to determine BoLA genotypes more efficiently (Table 2; Figure 2 and Figure S2). This targeted approach enabled sequencing of both DQA1 and DRB3 genes of up to 384 cows per flow cell. Given the stochastic nature of this approach, combining up to eight unique barcoded PCR amplicons per well (two genes, four cow barcodes), it was important to establish sequencing fidelity of allele identification prior to interpreting results. To validate the accuracy of this method, plasmids containing specific DRB3 alleles of interest were used as PCR templates and combined in homozygous and heterozygous fashion alone and in combination with BoLA amplicons from gDNA samples (Table S1).

All controls were properly identified with only one instance of barcode bleed-through (Well D6, Table S1) in which the *009:02 amplicon abundance led to *009:02 being called as the primary allele of the alternative barcode. However, *027:03 was found as the secondary allele for this barcode, indicating the stochastic nature, but high fidelity of the genotyping approach. It was theorized that as these barcoded PCR amplicons were produced from recombinant plasmid DNA template, and not gDNA extracts, the PCR efficiency was increased and resulted in the disproportionate amplification of a single allele. This is evident by the number of reads mapped to the *009:02 allele (44,567) compared to the *027:03 allele (2546). In all, this multiplex sequencing approach, capable of genotyping BoLA-DQA1 and BoLA-DRB3 genes of 384 cows per run, resulted in an 87% and 83% call rate for the DRB3 and DQA1 alleles, respectively. Although this call rate is lower than what is seen with other genotyping methods, it can be attributed to the high stringency used to make allele calls. To make a genotype call, we required a sample to have at least 100 reads mapping to a specific allele, consisting of at least 10% of the total reads, and variance less than two.

### 2.3. BLV Phenotype Associates with BoLA-DRB3 Genotype

**Figure 3:**
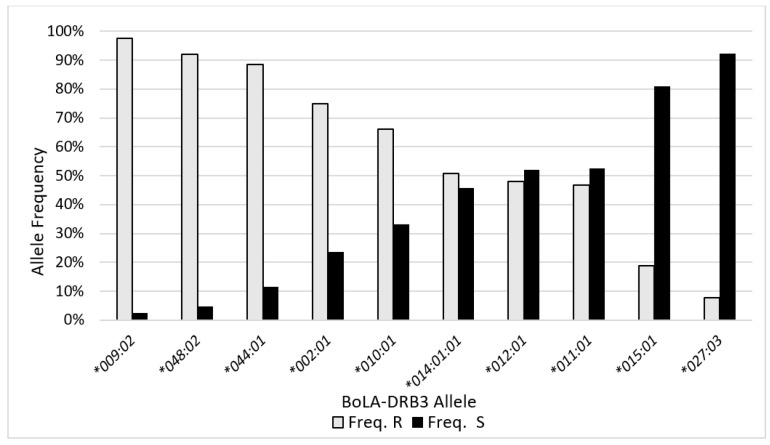
Comparison of BoLA-DRB3 allele frequencies between BLV resilient (R) and susceptible cows (S). Allele frequency in 335 resilient (grey) and 153 susceptible (black) cows were calculated for each BoLA-DRB3 allele that showed at least 4% frequency in the total population.

**Figure 4:**
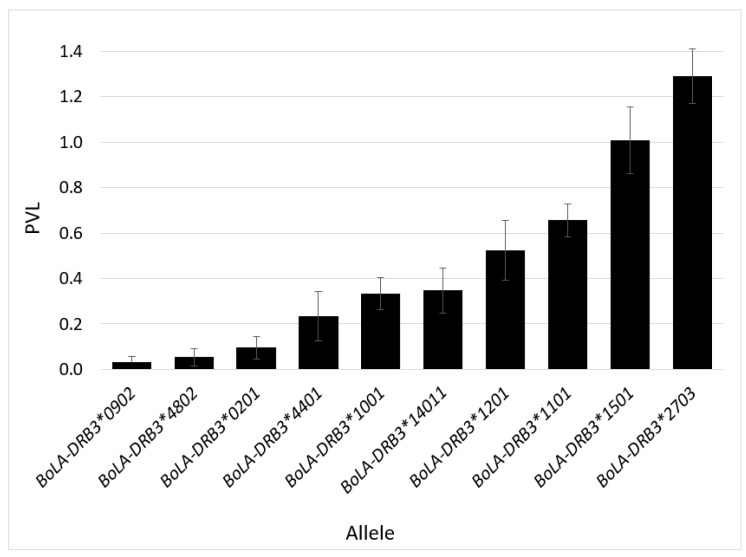
Comparison of average PVL per BoLA-DRB3 allele. Error bars represent 95% confidence interval.

Sequencing of BoLA-DQA1 and DRB3 revealed high amounts of immunogenetic homogeneity for both genes. We only identified eight different DQA1 alleles, with DQA1*014:01 and DQA1*001:01 found at frequencies of 48.8% and 19.2%, respectively (Table S2). Similarly, 18 DRB3 alleles were identified, with BoLA-DBR3*009:02 found at a frequency of 20% in our overall population (Table 3). However, our population was phenotypically selected for genotyping and contains more than double the number of resilient (*n* = 390) to susceptible (*n* = 168) cows. The most frequent allele found to be associated with BLV resilient cows was DRB3*009:02 (Figure 3). Of the total DRB3*009:02 alleles identified, 97% were possessed by BLV resilient cows. We saw similar trends with DRB3*048:02 (90% frequency in resilient cohort) and DRB3*044:01 (86% frequency in resilient cohort). Both DRB3*009:02 and *044:01 have previously been associated with BLV resilience [25,30]; however, this is one of the first reports demonstrating that DRB3*048:02 associates with BLV resilience. Conversely, BoLA-DRB3 alleles DRB3*015:01 and DRB3*027:03 were found at frequencies of 81% and 92%, respectively, within the phenotypically susceptible cohort, confirming this association with previous reports [19,24,30] (Figure 3; Table 3). The average PVL per DRB3 allele demonstrates that the two alleles most frequently observed in our resilient population (DRB3*009:02 and DRB3*048:02) also had the two lowest average PVL values (Figure 4). Similarly, the two most frequently found alleles in our susceptible population (DRB3*015:01 and DRB3*027:03) had the two highest average PVL values (Figure 4). It is important to note that the number of PVL observations included in the values represented in Figure 4 largely differs between alleles associated with the resilient versus susceptible phenotype. This is because most cows who possessed resilient alleles did not harbor detectable levels of the provirus, whereas cows with susceptible alleles had a positive PVL value each time they were tested.

### 2.4. BoLA-DRB3 Alleles Found to Be Associated with Differential BLV Phenotypes Are Genetically Related

**Figure 6:**
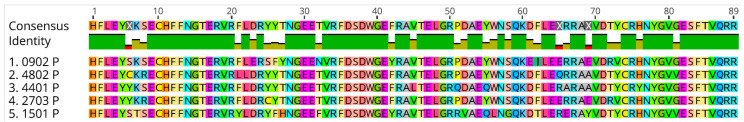
Amino acid sequence MUSCLE alignment of highly associated BLV resilient and susceptible BoLA-DRB3 alleles.

In response to the stark contrast between BLV phenotypes and their associated alleles, we assessed the genetic variation between the most frequently found resilient (*009:02, *044:01, and *048:02) and susceptible alleles (*015:01 and *027:03; Figure 5). Interestingly, the two most phenotypically distinct alleles, *009:02 and *027:03, are the most genetically related within commonly found DRB3 alleles in this study. Analysis of amino acid changes between the most frequent resilient and susceptible associated alleles reveals two regions within the second exon of the BoLA-DRB3 gene that have the most divergence, amino acids 6–8 and 65–69 (Figure 6). The most significant amino acid changes observed between *009:02 and *027:03 is a serine to tyrosine substitution at the 6th amino acid of the second exon of BoLA-DRB3. However, secondary structure analyses of these two highly related alleles did not result in significant alterations (data not shown).

## 3. Discussion

### 3.1. Disease Resilience, Tolerance, and Resistance

Disease resilience in livestock is defined as an animal’s ability to display negligible affects from a disturbance or the capability to return to their pre-exposure state [31]. This concept is similar to disease resistance and tolerance; however, crucial differences led us to define our population as BLV resilient. Disease resistance is an animal’s capability to limit its pathogen load by preventing infection overall or inhibiting pathogen replication [32,33]. Although our group has previously shown evidence for this phenomenon in BLV [27], we did not investigate whether resilient cows were able to inhibit within-host pathogen replication in this study. Furthermore, disease tolerance is described as the ability of an infected animal to limit the physiological damage caused by the pathogen load, without necessarily lowering the pathogen load [32,33]; this is also not an accurate characterization of our observations as resilient cows had undetectable levels of BLV provirus over time, and no additional physiological observations such as lymphocyte counts were obtained (Figure 1).

### 3.2. Longitudinal Diagnostic Outcomes for Phenotype Determination

Milk leukosis ELISA values of susceptible cows maintain a higher OD range, on average, than resilient cows, which agrees with the associated rise in PVL within susceptible cows over time (Figure 1). The apex of susceptible cow PVL values occurs around 60 months of age, followed by a decay of PVL values. This is likely due to selective culling of highly infected cows as this was the objective of the field trials [25,26] as well as the effect of immune responses in surviving cows, as older susceptible cow ELISA values continue to increase following PVL apex. Conversely, we observed a group of cows that maintain a relatively stable milk ELISA optical density scatter and negative PVL status throughout the duration of the study, which we deemed resilient to BLV progression. The phenotypic bifurcation of these two groups of seropositive cows is what prompted us to explore underlying immunogenetic associations within these cohorts.

### 3.3. Lack of Diversity in BoLA-MHC Class II Genes May Lead to Increased Disease Susceptibility

The European Molecular Biological Laboratory-European Bioinformatics Institute (EMBL-EBI) Major Histocompatibility Complex-Immuno Polymorphism Database (MHC-IPD) describes 384 BoLA-DRB3 alleles and 76 BoLA-DQA1 alleles [16]. Our study consisted of 558 Holstein cows from Midwestern dairies in the United States in which we identified 18 DRB3 and eight DQA1 alleles. For instance, almost 50% of all DQA1 alleles identified within this study were found to be *014:01, with the next most frequent allele being DQA1*001:01 at 19% (Table S2). This drastic reduction of allelic diversity within the MHC of modern dairy cattle undoubtedly contributes to a herd’s ability to combat the persistence of endemic pathogens such as BLV [6]. Importantly, the association between BoLA*009:02 and BLV resilience as well as DRB3*015:01 and *027:03 with BLV susceptibility has been confirmed within our phenotypically driven selection of cows. Genetic variability of the MHC region in humans has similarly been found to effect viral susceptibility, with the most studied example being HIV. MHC class I loci impacts the host management and acquisition of HIV, as well as viral load level and resistance to disease progression [34]. More specifically, almost 15% of the differences seen in viral load amongst HIV patients can be attributed to only two HLA genes [35].

When assessing the genotypic diversity of contemporary elite midwestern Holstein populations as compared to the 1960’s University of Minnesota West Central Research and Outreach Center dairy herd, there has been a drastic reduction in allelic diversity within the MHC loci [36]. Our data support this finding as out of 565 cows successfully genotyped from nine Midwestern US dairies, only 18 DRB3 and eight DQA1 alleles were found. In contrast, other studies investigating the diversity of this genomic region have found up to 71 distinct DRB3 alleles within 294 samples from Myanmar native cattle (11 alleles) and Holstein (33 alleles) [37]. This study helps exemplify the current understanding of immunogenetic relationships between disease resilience and the need to incorporate functional genomic elements such as MHC within selection indices to improve immunocompetence in dairy cattle.

### 3.4. BLV Phenotyping Prior to BoLA Genotyping Agrees with Current Literature

Immunogenetic associations between BoLA-DRB3 and BoLA-DQA1 and BLV disease status have been extensively characterized, initially through PCR-restriction fragment length polymorphism (PCR-RFLP) analysis [38] and more recently by PCR-targeted Sanger sequencing [14,15,18,19,22,30,39]. Therefore, we sought to extend these efforts and develop a higher throughput BoLA sequence-based typing approach using the Illumina MiSeq 2 × 250 sequencing platform. This work largely confirms the genotypic/phenotypic associations previously identified, while identifying a novel association of DBR3*048:02 with BLV resilience. It is important to note that the limited number of alleles found in 565 cows suggests the possibility of other DRB3 alleles may confer a similar immunologic advantage but are no longer circulating within US dairy herds, allowing for increased herd susceptibility with concomitant increases in genetic merit for milk production. Further mechanistic and genetic work is needed to elucidate why the DRB3*009:02 allele has such a strong association with BLV resilience.

### 3.5. BLV Phenotype Is Likely Affected by Various Genetic Factors

Comparison of the genetic relatedness of the most frequent alleles within each phenotypic group revealed resilient-associated alleles are closely related, however DRB3*027:03 was the most related allele to DRB3*009:02. This suggests that specific amino acid changes between these two alleles may pinpoint extracellular protein motifs responsible for the differential phenotypes observed. A similar effect has been shown in HIV1, in which it was found that a single amino acid change in an HLA MHC class I gene can have a significant effect on a patient’s progression to AIDS [40]. Furthermore, bovine research has shown possible associations between amino acid motifs in BoLA-DRB3 alleles and mastitis [41]. A recent study found variations in 17 antigen recognition sites within exon 2 of the BoLA-DRB3 gene, with 11 amino acids associating with BLV resistance, and 11 conferring BLV susceptibility [30]. This suggests that either the polarity, charge of amino acids in binding pockets, or both, may confer BLV resistance or susceptibility. However, looking into protein electrostatic interactions was beyond the scope of this study, and more in-depth mutational and protein confirmational studies in the context of BoLA DQA1/DRB3 heterodimers are needed to gain more insights into how these proteins may interact with BLV antigens.

Though the structural differences amongst BoLA MHC II alleles may affect BLV phenotype, additional genomic loci undoubtedly play a role. In HIV, two coreceptors (CCR5 and CXCR4) were found to effect disease progression such that individuals who lack these genes display HIV resistance [42]. A similar phenomenon may be taking place with BLV and the SLC7A1 gene. The BLV Env protein binds to the cationic amino acid transporter 1 (CAT1)/solute carrier family 7 member 1 (SLC7A1), and although it is unknown if CAT1/SLC7A1 is a receptor for BLV in vivo, cells with undetectable CAT1 levels were found to be resistant to BLV [43]. Likewise, cells with overexpressed CAT1 levels became susceptible to BLV infection [43]. In addition to CAT1/SLC7A1, there is evidence that identifies the CD209 protein as a potential receptor for BLV [44,45]. CD209 is expressed in B-cells [45] and polymorphisms in its promoter has been linked to HTLV [46]. More recently, a method has been developed to knock out the CD209 protein in hopes of creating cells that display resistance to BLV [44]. Although a definitive BLV receptor has not been identified in vivo, these two examples further suggest the influence of genetic regions outside aside from BoLA genes that influence BLV phenotype. Going forward, it will be important to determine other genetic elements that may segregate with BoLA MHC II alleles, specifically within other immune loci on the bovine 23rd chromosome, to aid in the understanding between bovine humoral immunity and BLV resilience.

## 4. Materials and Methods

### 4.1. Sample Collection, Diagnosis, and Phenotype Determination

From fall of 2016 to fall of 2020, annual or biannual (herd dependent) DHI milk samples were collected and screened for anti-BLV antibodies via ELISA (CentralStar Cooperative, Grand Ledge, MI, USA). Subsequently, blood was collected by coccygeal venipuncture into K_2_ EDTA Vacutainer tubes from all BLV ELISA positive cows (Table 1). Samples for sequencing were selected based on differential BLV phenotypes, identified through our testing approach. BLV resilient cows were defined as persistently ELISA positive without detectable levels of provirus, while BLV susceptible cows remained both ELISA positive and PVL positive (Figure 1).

### 4.2. Detection of Anti-BLV gp51 Antibody via Enzyme-Linked Immunosorbent Assay (ELISA)

Individual DHI milk samples were tested using a modified ELISA test (IDEXX Laboratories, Inc., Westbrook, ME, USA) to identify the BLV serostatus of the cows at the time of bleeding. In short, milk samples were diluted in sample buffer and pipetted into 96-well plates coated with BLV-GP51 antigen. Horseradish peroxidase-labeled bovine anti-immunoglobulin was added followed by incubation at room temperature for 30 min. Plates were washed after each incubation and before adding the enzyme-substrate and then incubated for 10 min before being stopped by adding 0.5 N (sulfoamino) oxidanide (H_2_NO_4_S). Results were reported as corrected 450 nm optical density (OD) measurements with a corrected OD > 0.3 being considered antibody positive.

### 4.3. DNA Extraction and BLV Proviral Load Determination

DNA was extracted from whole blood using a modified DNeasy Blood and Tissue gDNA extraction kit protocol (Qiagen, Germantown, MD, USA). Briefly, 200 µL of fresh whole blood from K_2_ EDTA Vacutainer tubes was incubated with 40 µL of protease K and 219 µL of Buffer AL at 56 °C for 10 min prior to the addition of 219 µL of 100% ethanol and mixed via pipetting and transferred to DNeasy spin column, while the remainder of the DNA isolation was performed per the manufacturer’s instructions. BLV proviral load was quantified using the BLV SS1 qPCR PVL Assay (CentralStar Cooperative, East Lansing, MI, USA). PVL values were calculated by deriving BLV and bovine DNA copy number via standard curve qPCR machine calibration (Table S2) and dividing the number of BLV copies by the number of bovine DNA copies and expressed as a ratio of BLV: bovine DNA content.

### 4.4. Next Generation Sequencing (NGS) of the BoLA-DRB3 Gene

BoLA haplotypes were determined using a novel multiplex next-generation sequence based typing (NGS-SBT). Using target specific primers developed by Eijk et al., 1992 [29], amplicons of the second exon of the DRB3 and DQA1 genes were produced via end point PCR. These primers were modified to include animal and gene specific TruSeq indexes enabling up to four cows and two genes per cow in each well. Universal Common sequence (Fluidigm, San Francisco, CA, USA) tag was added to the 5′ end needed for sequencing on the Illumina MiSeq platform for sequencing plate well-specific indexes (Table 2). All amplicons were visualized using electrophoretic analysis to confirm correct amplicon size prior to library preparation and sequencing. Three separate sequencing runs were completed with 96–384 cows per plate for a total number of 565 cows. All amplicons were sequenced via targeted 2 × 250 bp paired end format using a MiSeq v2 500 cycle flow cell. All library preparation and sequencing were performed by The Research Technology Support Facility (RTSF) at Michigan State University.

### 4.5. Bioinformatics

Reads were trimmed for quality using trimmomatic (v0.36) with default settings (Bolger). Trimmed reads were aligned to a reference genome made up of known DQA and DRB3 alleles using bwa mem (v0.7.17-r1198-dirty) with default settings [47]. Counts of reads aligning to each reference sequence with MAPQ of at least 10 were obtained with samtools (v1.7) [48]. Variant calling was performed using freebayes (v1.3.2-46-g2c1e395) with default settings [49]. Variants with QUAL < 20 were discarded from further analysis. Genotype calls were determined via a heuristic approach leveraging the count of reads mapping to a given allele and the number of variants identified within that allele. Our criteria for making a genotype call required a sample to have at least 100 reads mapping to a specific allele, consisting of at least 10% of the total reads per barcode, and variance less than 2. A cow was called heterozygous if the two alleles evenly split the percent reads mapping (50:50, 60:40). A cow was deemed homozygous if at least 80% of the reads mapped to one allele. If the number of reads split 70:30, the allele with the majority reads was called and the second was deemed low reads and remained uncalled.

### 4.6. Sequencing Controls

Allele controls were chosen based on their genetic relatedness to the target allele, BoLA-DRB3*009:02. All plasmids were cloned into a pUC-57 vector (Addgene, Watertown, MA, USA), transformed using DH5-α chemically competent cells (ThermoFisher, Waltham, MA, USA), and digested using ScaI for template linearization (Table S1, Figure S2). The DRB3 allele was amplified using the same primers used to create amplicons in the genomic DNA samples. Samples were multiplexed into the sequencing plate, for a total of two samples per well. Controls were added to the sequencing plate in homozygous genotypes and added to wells with another control or genomic DNA. Genotypes were called for the controls using the same criteria used to determine the sample genotypes (Table 2).

### 4.7. Phylogenetics and Amino Acid Analysis

Phylogenetic analysis was performed to examine the evolutionary genetic differences between the alleles identified in this study. Nucleotide and amino acid sequences corresponding to the discovered alleles were gathered from the European Molecular Biological Laboratory-European Bioinformatics Institute (EMBL-EBI) Major Histocompatibility Complex-Immuno Polymorphism Database (MHC-IPD). These sequences were aligned using MUSCLE with 64 iterations [50]. A neighbor-joining method with the maximum composite likelihood model was used to construct the phylogenetic tree with 1000-bootstrap replications in MEGAX. The tree was rooted at the midpoint with an increasing node order and was visualized in FigTree (version 1.4.3). Geneious Prime 11.0.11+9 was used to align the amino acid sequences for mutation analysis between the most common alleles found within the identified resilient and susceptible populations.

## Figures and Tables

**Figure 5:**
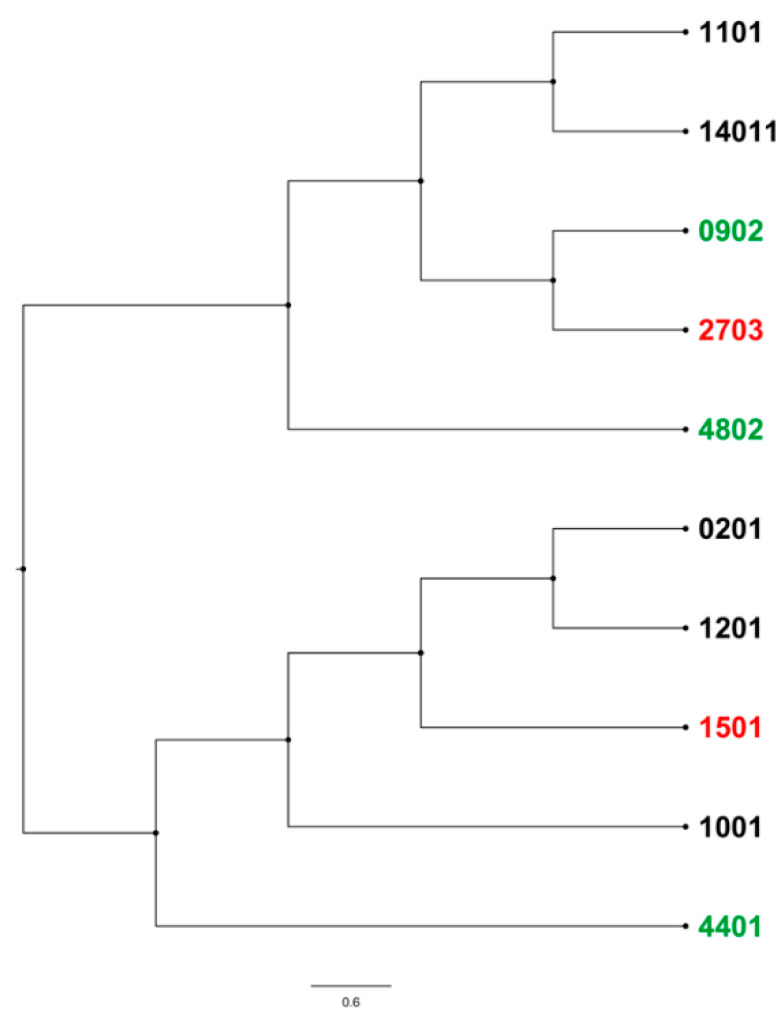
Molecular phylogeny of frequently found BoLA-DRB3 alleles. Neighbor-joining tree with maximum composite likelihood model. Green alleles indicate DRB3 alleles associated with resilience whereas red alleles show association with susceptibility.

**Table 1:** BLV eradication field trial herds and cow numbers.

	Timepoints				
	2016	2017	2018	2019	Total
# Farms	6	6	9	9	9
# ELISA	80	866	6633	6854	14,433
# PCR	65	30	1655	2335	4085
# Genotyped Animals		95	95	384	574

Cows from nine Midwestern dairy farms were screened. Herd sizes ranges were <300 (*n* = 3), 500–1500 (*n* = 3), 2000–3500 (*n* = 2), and <6000 (*n* = 1) cows.

**Table 2:** Multiplex next generation sequence based typing (NGS-SBT) approach.

Primer Name	Fluidigm CS Sequence	TruSeq Index	Gene-Specific Primer Sequence
BoLA-DRB3.1 F	ACACTGACGACATGGTTCTACA	TCGTGGAGCG	ATCCTCTCTCTGCACGAGATTTCC
BoLA-DRB3.4 F	ACACTGACGACATGGTTCTACA	TGCCTGGTGG	ATCCTCTCTCTGCACGAGATTTCC
BoLA-DRB3.12 F	ACACTGACGACATGGTTCTACA	GTGTGGCGCT	ATCCTCTCTCTGCACGAGATTTCC
BoLA-DRB3.20 F	ACACTGACGACATGGTTCTACA	CGCACATGGC	ATCCTCTCTCTGCACGAGATTTCC
BoLA-DRB3 R	TACGGTAGCAGAGACTTGGTCTTCGCCGCTGCACAGTGAAACTCTC		

Our gene specific primers, developed by Eijk et al., 1992 [29] were modified for multiplexing using the TruSeq adapter sequencing kit to add one of four unique 10 bp animal barcodes and a 22 bp Illumina Universal Common Sequence (Fluidigm, San Francisco, CA, USA) tag to the 5′ end needed to sequence on the Illumina MiSeq Platform. Using this approach, we included four animals and two gene amplicons per well of a 96-well sequencing plate.

**Table 3:** Allelic frequency of BoLA-DRB3 alleles called within resilient (R) and susceptible (S) cohorts found in >2% of the cows included in the study.

BoLA-DRB3 Allele	Total No.	Total Freq.	Freq. R	Freq. S
*009:02	199	20.2%	97.5%	2.5%
*011:01	171	17.4%	46.8%	52.6%
*010:01	124	12.6%	66.1%	33.1%
*002:01	72	7.3%	75.0%	23.6%
*048:02	62	6.3%	91.9%	4.8%
*014:01:01	57	5.8%	50.9%	45.6%
*044:01	52	5.3%	88.5%	11.5%
*012:01	50	5.1%	48.0%	52.0%
*027:03	39	4.0%	7.7%	92.3%
*015:01	37	3.8%	18.9%	81.1%
*016:01	35	3.6%	85.7%	11.4%
*007:01	33	3.4%	78.8%	18.2%
*006:01	27	2.7%	92.6%	7.4%
*001:01	9	0.9%	33.3%	66.7%
*037:01	6	0.6%	83.3%	16.7%
*018:01	6	0.6%	50.0%	50.0%
*008:01	4	0.4%	50.0%	50.0%
*061:01	1	0.1%	0.0%	100.0%

## Data Availability

Data is contained within the article and Supplementary Materials.

## Supplementary Materials

The following supporting information can be downloaded at: www.mdpi.com/article/10.3390/pathogens11010104/s1, Figure S1: Calibration of the SS1 BLV proviral load assay, Figure S2: Molecular cloning of specific BoLA-DRB3 alleles into puC-57 plasmids for sequencing controls, Table S1: BoLA-DRB3 plasmid control sequencing results, Table S2: Allelic frequency of BoLA-DQA1 of total population and within phenotypic cohorts.
