# Supplementary material for: Phenotypic Selection of Dairy Cattle Infected with Bovine Leukemia Virus Demonstrates Immunogenetic Resilience through NGS-Based Genotyping of BoLA MHC Class II Genes
Source: Pathogens. 2022 Jan 15;11(1):104. doi: 10.3390/pathogens11010104 (PMC8779071; doi:10.3390/pathogens11010104)
Supplement: Supplementary file 1 [file pathogens-11-00104-s001.zip › pathogens-1511785-supplementary.pdf]

SUPPLEMENTAL

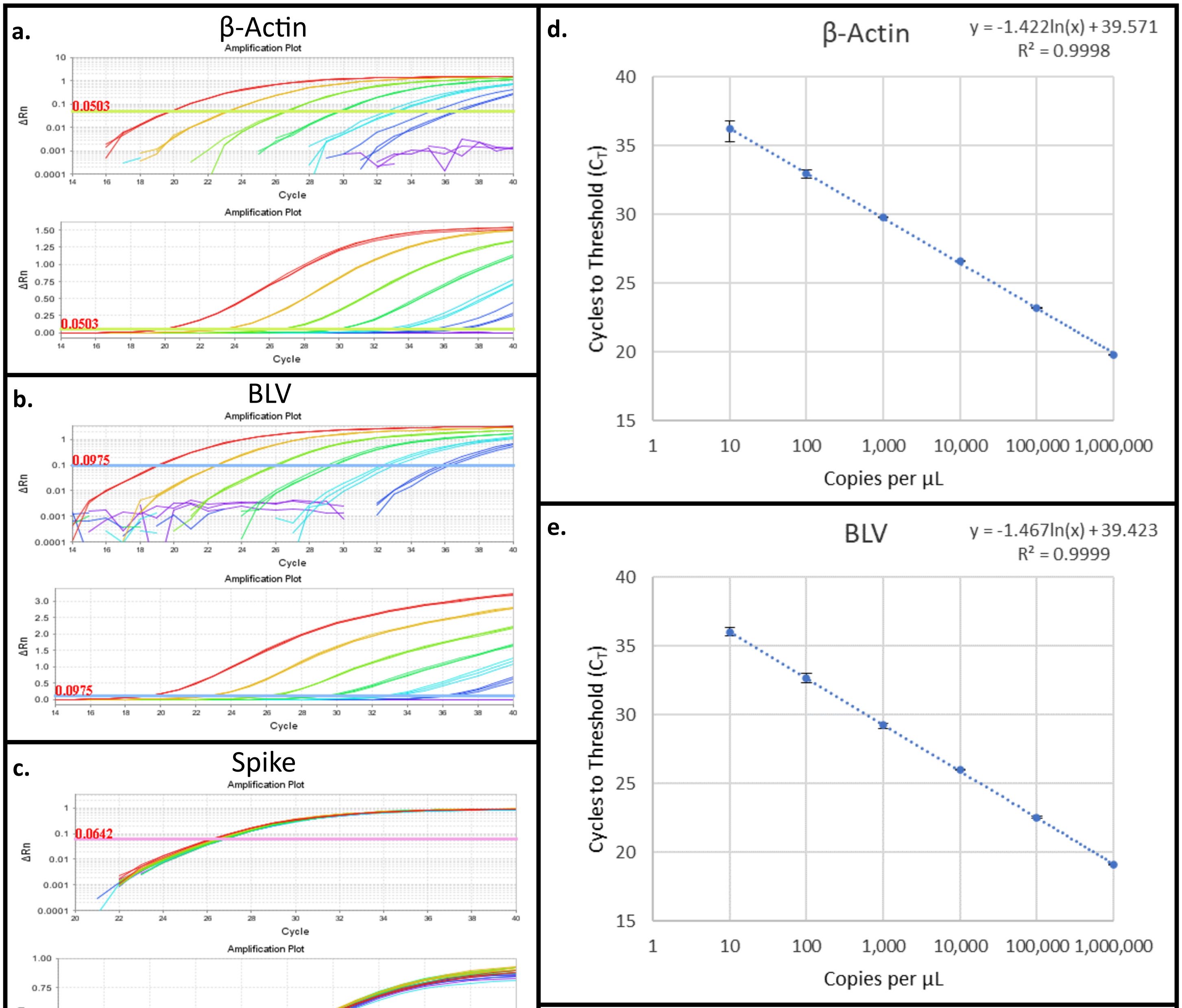

f.

$$PVL = (e^{(BLV\ CT-39.423)/-1.467})/(e^{(\beta\text{-Actin}\ CT-39.571)/-1.422})$$

Figure S1: Calibration of the SS1 BLV proviral load assay. DNA standards were produced through traditional molecular cloning techniques where PCR target templates were cloned into the puC57 DNA Vector, linearized via ScaI restriction enzyme and subsequently quantified via serial dilution and ddPCR. Original aliquots were normalized to $1x10^6$ copies/µL with Tris-EDTA pH 8.0 and 5 ng/µL of polyA carrier RNA (Thermo Fisher) prior to PCR calibration. 4x20 µL aliquots of this stock were prepared for both β-Actin and BLV. Microcentrifuge tubes for each target were labeled with dilution (10^5, 10^4, 10^3, 10^2, 10^1) and loaded with 45 µL TE. Serial dilutions were performed by adding 5 µL of previous dilution, vortexing and spinning down between each. The DNA standards were subjected to SS1 PCR in triplicate; analysis thresholds were set at 3% of amplification curve plateau for both β-Actin (a) and BLV (b), and at 7% for Spike (c). Average CT results for β-Actin (d) and BLV (e) were graphed in scatter plots to generate a line of regression for each, relating CT to copies of target sequence per 1 µL. These regression equations were used to generate the formula (f) used to calculate proviral load (PVL), the relative concentration of BLV proviral DNA to bovine DNA amplified in the sample.

Table S1: BoLA-DRB3 Plasmid control sequencing results. Plasmids containing specific DRB3 alleles were used as PCR templates for producing barcoded DRB3 amplicons and sequenced alone and in combination with PCR amplicons from animal gDNA templates. None of the gDNA-derived amplicons from animals were included in this study.

Well	Well Input	DRB3 Control Name	TruSeq ID	Control Primary Allele			Control Secondary Allele			Plasmid Final Call
				Read Depth	% Mapped	Allele Call	Read Depth	% Mapped	Allele Call	
A10	Plasmid/gDNA	*027:03	TGCCTGGT**GG**	3,858	59%	2703	1,116	17%	1801	2703/Low Reads
H9	Plasmid/gDNA	*027:03	TGCCTGGT**GG**	4,708	48%	2703	<100	<10%	0902	2703/Low Reads
D6	Plasmid/Plasmid	*027:03	TGCCTGGT**GG**	4,317	62%	0902	**2,546**	**36%**	**2703**	0902/2703
A11	Plasmid/gDNA	*009:02	TGCCTGGT**GG**	36,456	97%	0902	<100	<10%	0902	0902/0902
H10	Plasmid/gDNA	*009:02	TGCCTGGT**GG**	58,254	95%	0902	706	<10%	0902	0902/0902
D6	Plasmid/Plasmid	*009:02	TCGTGGAG**CG**	44,567	99%	0902	243	<10%	0902	0902/0902
H12	Plasmid/Plasmid	*009:02	TCGTGGAG**CG**	98,744	99%	0902	<100	<10%	0902	0902/0902
A5	Plasmid/gDNA	*009:02	TGCCTGGT**GG**	55,874	94%	0902	<100	<10%	0902	0902/0902
B11	Plasmid/gDNA	*009:02	TGCCTGGT**GG**	53,389	88%	0902	1665	<10%	0902	0902/0902
H11	Plasmid/gDNA	*009:02	TGCCTGGT**GG**	24,554	92%	0902	<100	<10%	0902	0902/0902
H12	Plasmid/Plasmid	*009:02	TGCCTGGT**GG**	99,565	99%	0902	<100	<10%	0902	0902/0902
D11	Plasmid/gDNA	*009:03	TCGTGGAG**CG**	70,145	94%	0903	1,090	<10%	0903	0903/0903
A8	Plasmid/gDNA	*009:03	TGCCTGGT**GG**	52,986	92%	0903	865	<10%	0903	0903/0903
A9	Plasmid/gDNA	*009:04	TCGTGGAG**CG**	<100	<10%	2601	<100	<10%	1801	low reads/low reads
H8	Plasmid/gDNA	*009:04	TGCCTGGT**GG**	2,402	<10%	1801	<100	<10%	0901	low reads/low reads

Well	Animal ID	TruSeq ID	Control Primary Allele			Control Secondary Allele			gDNA Final Call
			Read Depth	% Mapped	Allele Call	Read Depth	% Mapped	Allele Call	
A10	DOW 151	TCGTGGAG**CG**	26,210	93%	1801	1,056	<10%	1101	1801/1801
H9	DOW 3020	TCGTGGAG**CG**	35,021	96%	2601	<100	<10%	3601	2601/2601
D6	N/A								
A11	DOW 813	TCGTGGAG**CG**	1,789	23%	0902	<100	<10%	0201	0902/Low Reads
H10	DOW 3025	TCGTGGAG**CG**	23,500	53%	2601	18,105	41%	1801	2601/1801
D6	N/A								
H12	N/A								
A5	DOW 1412	TCGTGGAG**CG**	25,003	84%	0201	3,870	13%	0902	0201/0201
B11	DOW 051	TCGTGGAG**CG**	20,749	41%	1801	3,448	<10%	0902	1801/Low Reads
H11	DOW 3029	TCGTGGAG**CG**	15,461	95%	2601	620	<10%	0902	2601/2601
H12	N/A								
D11	SIKMA 9311	TGCCTGGT**GG**	21,358	52%	3301	15,113	37%	1801	3301/1801
A8	50-P4	TCGTGGAG**CG**	17,614	53%	3301	11,472	34%	1801	3301/1801
A9	SM 513	TGCCTGGT**GG**	139	10%	14011	<100	<10%	1801	14011/Low Reads
H8	DOW 3016	TCGTGGAG**CG**	32,417	83%	1801	113	<10%	0201	1801/1801

Table S2. Allelic Frequency of BoLA-DQA1 of total population and within phenotypic cohorts.

BoLA-DQA Allele	Total No.	Total Freq
*014:01	452	48.4%
*001:01	178	19.1%
*010:02	144	15.4%
*012:04	64	6.9%
*003:01	62	6.6%
*001:03	20	2.1%
*012:01:01	7	0.7%
*003:01	7	0.7%

BoLA-DQA Allele	Freq. R	Freq. S
*003:01	90.3%	6.5%
*013:01	85.7%	14.3%
*014:01	72.8%	25.0%
*001:03	65.0%	35.0%
*010:02	62.5%	37.5%
*001:01	56.2%	42.7%
*012:04	21.9%	78.1%
*012:01:01	0.0%	100.0%

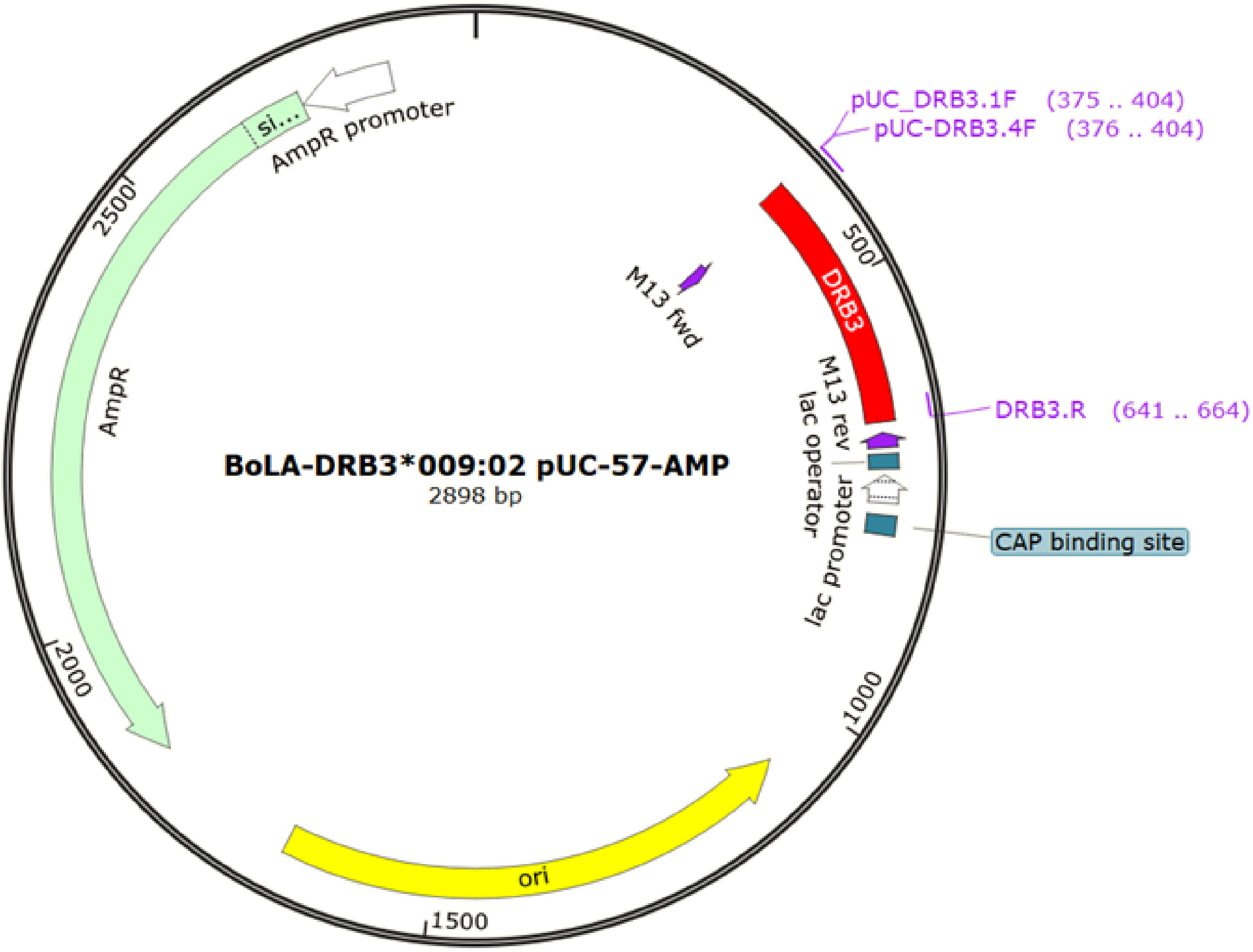

Figure S2. Molecular cloning of specific BoLA-DRB3 alleles into puC-57 plasmids for sequencing controls. Barcoded forward primers and universal reverse primers were used to amplify the target insert prior to library preparation and sequencing.

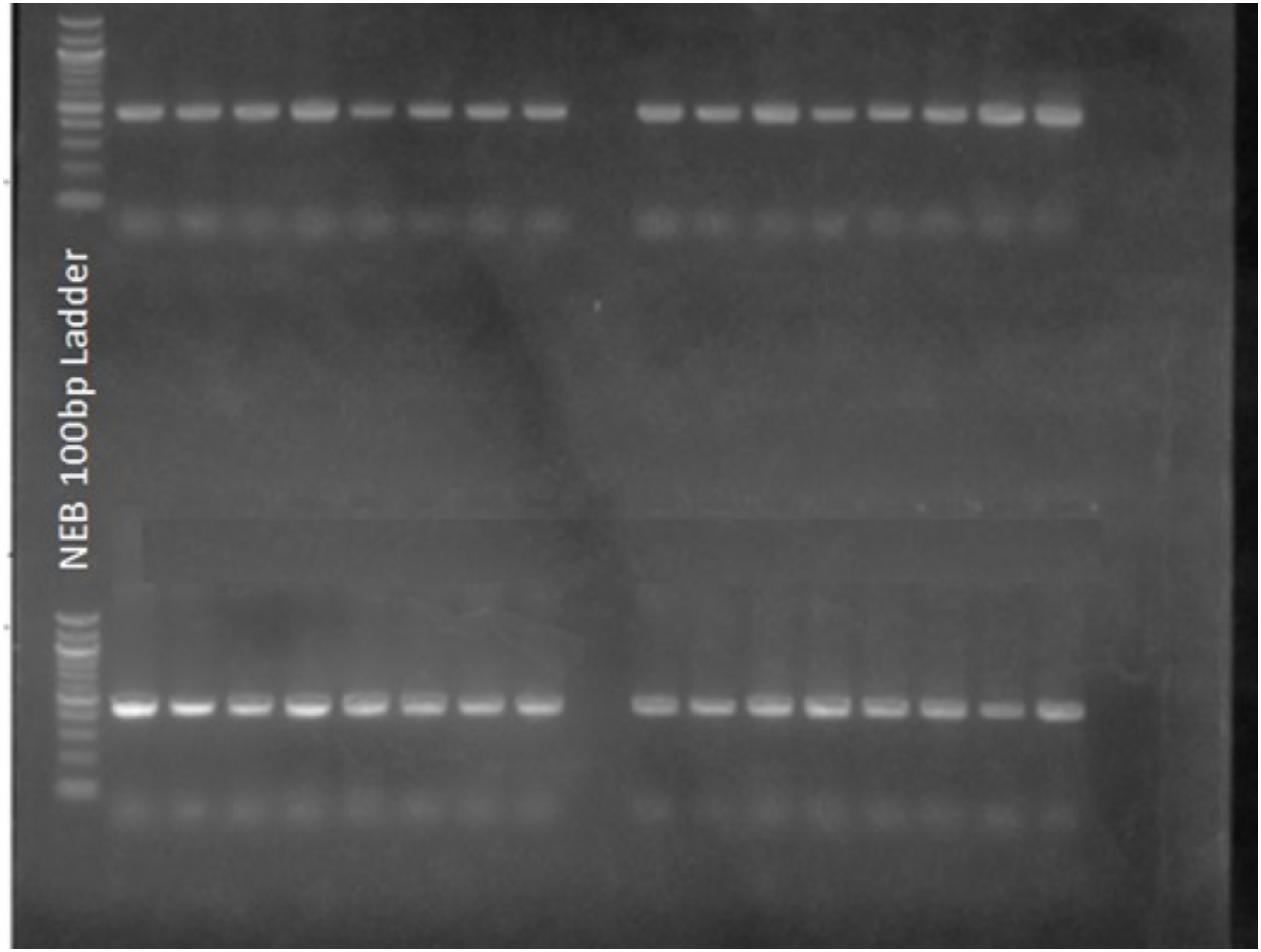

Figure S3. Example gel electrophoresis of BoLA-DRB3 barcoded amplicons used to evaluate PCR reactions prior to library preparation.
